# A multidisciplinary pulmonary embolism response team (PERT): first experience from a single center in Germany

**DOI:** 10.1007/s00392-023-02364-4

**Published:** 2023-12-19

**Authors:** Ingo Sagoschen, Barbara Scibior, Ioannis T. Farmakis, Karsten Keller, Dirk Graafen, Eva-Verena Griemert, Markus Vosseler, Hendrik Treede, Thomas Münzel, Maike Knorr, Tommaso Gori, Stavros Konstantinides, Lukas Hobohm

**Affiliations:** 1 Department of Cardiology, University Medical Center of the Johannes Gutenberg-University Mainz, Mainz, Germany; 2 Center for Thrombosis and Hemostasis (CTH), University Medical Center of the Johannes Gutenberg University Mainz, Mainz, Germany; 3 Medical Clinic VII, University Hospital Heidelberg, Heidelberg, Germany; 4 Department of Diagnostic and Interventional Radiology, University Medical Center of the Johannes Gutenberg-University Mainz, Mainz, Germany; 5 Department of Anesthesiology, University Medical Center of the Johannes Gutenberg-University Mainz, Mainz, Germany; 6 Department for Cardiac and Vascular Surgery, University Medical Center Mainz of the Johannes Gutenberg-University Mainz, Mainz, Germany; 7 Department of Cardiology, Democritus University of Thrace, Komotini, Greece

**Keywords:** Pulmonary embolism, Pulmonary embolism response team, Advanced therapies, Catheter-directed treatment, Systemic thrombolysis

## Abstract

**Background:**

Over the last few years, the concept of multidisciplinary pulmonary embolism response teams (PERTs) has emerged to encounter the increasing variety and complexity in managing acute pulmonary embolism (PE).

**Purpose:**

To investigate PERT's composition and added clinical value in a university center in Germany.

**Methods:**

Over 4 years (01/2019–11/2022), patients with confirmed PE were enrolled in a prospective single-center cohort study (PERT Mainz). We investigated the composition of PERT and compared, after propensity score matching, patients with acute PE before and after the initiation of PERT at our Medical University Centre. The primary outcome was in-hospital PE-related mortality.

**Results:**

From 2019 to 2022, 88 patients with acute PE with a PERT decision were registered. Of those, 13 (14.8%) patients died during the in-hospital stay. Patients evaluated by a PERT had a median age of 68; 48.9% were females, and 21.7% suffered from malignancy. Right ventricular dysfunction was present in 76.1% of all patients. In total, 42.0% were classified as intermediate–high-risk PE and 11.4% as high-risk PE. First PERT contact mainly originated from emergency departments (33.3%) and intensive care units (30.0%), followed by chest pain units (21.3%) and regular wards (12.0%). The participation rate of medical specialties demonstrated that cardiologists (100%) or cardiac/vascular surgeons (98.6%) were included in almost all PERT consultations, followed by radiologists (95.9%) and anesthesiologists (87.8%). Compared to the PERT era, more patients in the pre-PERT era were classified as simplified pulmonary embolism severity index (sPESI) ≥ 1 (78.4% vs 71.6%) and as high-risk PE according to ESC 2019 guidelines (18.2% vs. 11.4%). In the pre-PERT era, low- and intermediate-low patients with PE received more frequently advanced reperfusion therapies such as systemic thrombolysis or surgical embolectomy compared to the PERT era (10.7% vs. 2.5%). Patients in the pre-PERT were found to have a considerably higher all-cause mortality and PE-related mortality rate (31.8% vs. 14.8%) compared to patients in the PERT era (22.7% vs. 13.6%). After propensity matching (1:1) by including parameters as age, sex, sPESI, and ESC risk classes, univariate regression analyses demonstrated that the PE management based on a PERT decision was associated with lower risk of all-cause mortality (OR, 0.37 [95%CI 0.18–0.77]; *p = *0.009). For PE-related mortality, a tendency for reduction was observed (OR, 0.54 [95%CI 0.24–1.18]; *p = *0.121).

**Conclusion:**

PERT implementation was associated with a lower risk of all-cause mortality rate in patients with acute PE. Large prospective studies are needed further to explore the impact of PERTs on clinical outcomes.

**Graphical abstract:**

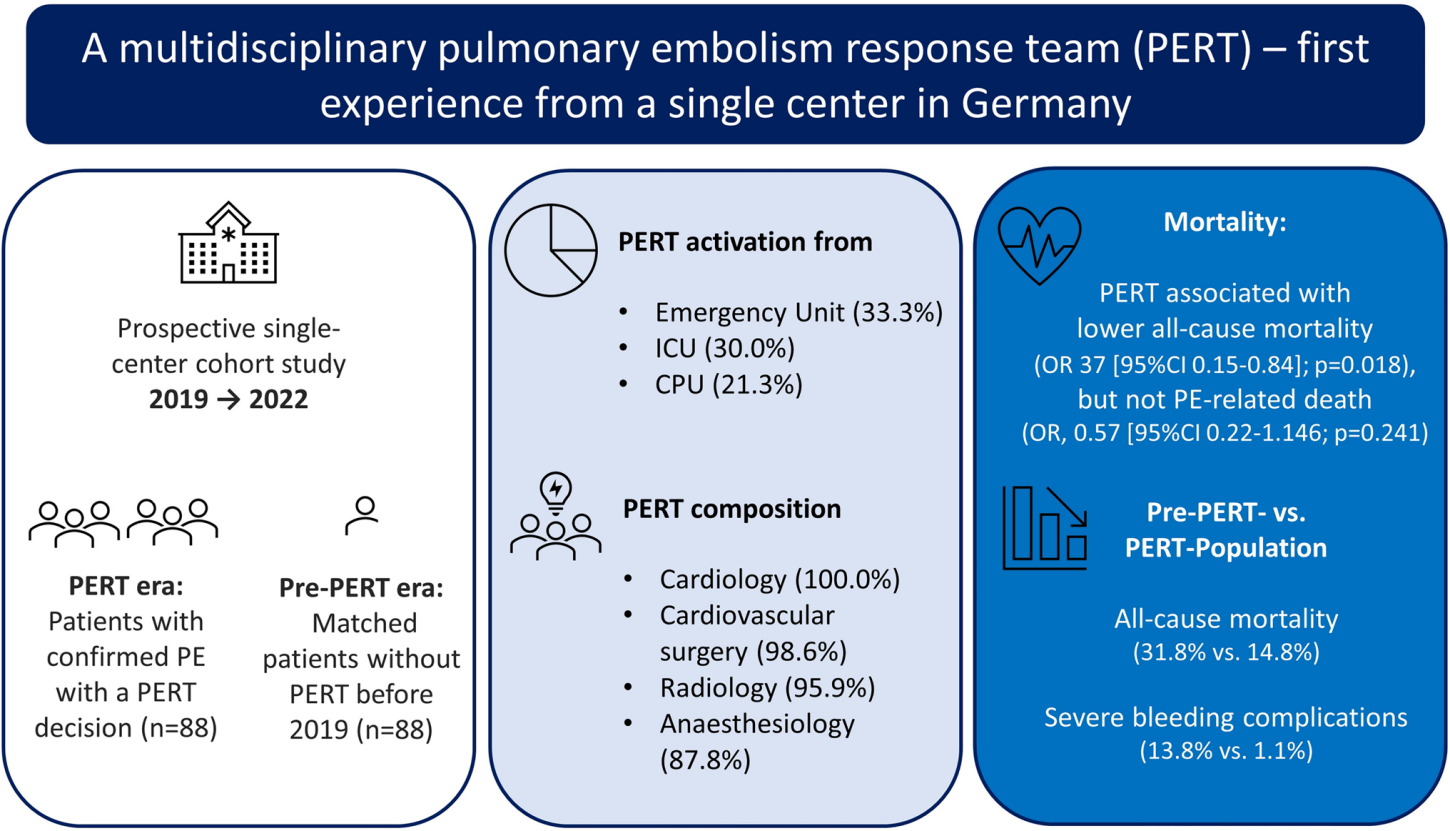

**Supplementary Information:**

The online version contains supplementary material available at 10.1007/s00392-023-02364-4.

## Introduction

Acute pulmonary embolism (PE) is a common, often undiagnosed, but potentially life-threatening condition caused by the obstruction of pulmonary arteries by thromboembolic evolving mainly from pelvic and lower limb veins [1]. PE can lead to various clinical presentations, from asymptomatic to severe hemodynamic instability, shock, and sudden cardiac arrest [2]. Early and accurate diagnosis, risk stratification, and adapted treatment regimens are crucial to reduce associated morbidity and mortality. However, the management of PE can be challenging, especially in patients with high-risk or intermediate-risk PE. The risk of bleeding frequently limits the treatment options of patients due to underlying comorbidities and risk factors. These patients require a multidisciplinary approach, individualized therapies, and specialized care [3].

Pulmonary embolism response teams (PERTs) have emerged as a new paradigm in managing acute PE, aiming to provide immediate and coordinated care to patients with PE at higher risk in light of an evolving complex armamentarium of advanced treatment options, such as catheter-based therapies [4]. Additionally, PERT can bridge definitive therapy by deciding on temporary extracorporeal membrane oxygenation for cardiopulmonary support for patients not candidates for immediate reperfusion [5]. PERTs are multidisciplinary teams of experts from various specialties, including cardiology, pulmonology, radiology, cardiac and vascular surgery, hematology, and critical care medicine [6]. The main goal of PERTs is to optimize and accelerate the diagnosis, risk stratification, and treatment of patients with acute PE, using a collaborative and evidence-based approach. The importance of these multidisciplinary teams in managing acute PE has been recognized recently by certain medical societies, e.g., the European Society of Cardiology (ESC) [3].

Literature regarding PERT implementation along with guidelines change is limited. Several single-center reports and studies, mainly from North America, show inconsistent results. Additionally, little is known about the experience of this multidisciplinary treatment approach in Germany. Thus, we sought to investigate the first single-center experience from a university hospital in Germany, which implemented PERT a few years ago.

## Materials and methods

### Patient cohort and study design

Patients aged ≥ 18 years with confirmed acute PE and a protocol of pulmonary embolism response team (PERT) were included in this study. According to the PERT protocol, this includes patients stratified as “high risk” and “intermediate high risk” using the classification provided by the ESC guidelines and all patients with lower risk PE and complicating factors (e.g., neurosurgical patients) [7, 8].

The study was performed as an observational single-center prospective cohort study (Pulmonary Embolism Registry Mainz, PERT Mainz) between January 2019 and November 2022. In order to perform a propensity score matching, a retrospective control cohort included all patients with acute PE (based on ICD 10 code) treated on intermediate-care or intensive care units from January 2017 until December 2018 (Pre-PERT Fig. S1) was analyzed. Patients were stratified post hoc in risk classes according to the sPESI [9] and the algorithm proposed by the 2019 ESC guidelines [10].

The outcomes of interest included PE-related death, in-hospital mortality, and bleeding events. Major bleeding was defined as fatal and/or symptomatic bleeding in a critical area or organ and/ or bleeding causing a fall in hemoglobin level of ≥ 2 g/dl or transfusion of ≥ 2 units of erythrocyte concentrates according the definition of the International Society of Thrombosis and Haemostasis (ISTH). All patients were followed up during the in-hospital stay. Treatment decisions were made by the physicians caring for the patient according to current guidelines and were not predefined or influenced by the study protocol. Study results were not communicated to the clinicians and thus not used to guide patient management or monitor treatment effects at any time during the observation. The study protocol was conducted following the amended Declaration of Helsinki and was approved by the local independent Ethic Committees at the study center.

### Statistical analysis

The Fisher´s exact test or the chi-square test was used to compare categorical variables, which are expressed as absolute number or percentage. Continuous variables were found not to follow a normal distribution when tested with the modified Kolmogorov–Smirnov test (Lilliefors test); therefore, these variables are expressed as medians with the corresponding interquartile range (IQR) and compared using the unpaired Mann–Whitney *U* test. We investigated the composition of PERT and compared, after propensity score matching, patients with acute PE prior and after the formation of PERT at our institution. Parameters, such as sex, age, sPESI, and ESC risk classes, included as matching variables (Fig. S1). The prognostic relevance of the PERT era or not as well as single predictors concerning study outcomes was then tested using univariable logistic regression analysis and presented as odds ratios (OR) with corresponding 95% confidence intervals (CIs). A two-sided significance level of *α* < 0.05 was defined as appropriate to indicate statistical significance. Statistical analyses were performed using the SPSS software (version 21.0, SPSS Inc., Chicago, Illinois, USA) and R (version 4.2.2., R Foundation for Statistical Computing, Vienna, Austria).

## Results

### PERT era: Baseline characteristics and risk stratification

Overall, 88 patients with acute PE were included in the PERT Mainz registry. The gender distribution was almost equal, with a median age of 68 years (Table 1). Cardiovascular comorbidities were common in these patients: Overall, 68.2% with arterial hypertension, 14.6% were diagnosed with coronary artery disease, 11.0% with diabetes, 23.9% with chronic cardiac or pulmonary disease. Risk factors for venous thromboembolism (VTE), such as recent hospitalization or immobilization, were present in 30.5% of all cases as well as previous VTE events in 14 (17.3%) patients and a history of malignancy in 18 (21.7%) patients (Table 1). Right ventricular dysfunction was present in 67 (76.1%) patients. In total, 42.0% were classified as intermediate–high-risk PE and 11.4% as high-risk PE. During the in-hospital stay, 13 (14.8%) patients died.

**Table 1:** Baseline characteristics of non-survivors and survivors (PERT era)

	All patients (*n = *88)	Non-Survivors (*n = *13; 14.8%)	Survivors (*n = *75; 85.2%)	*p* value
Sex (male)	45 (51.1%)	7 (53.8%)	38 (50.7%)	1.000
Median Age (IQR)	68 (58–76)	67 (58–72)	69 (58–79)	0.488
Age above 80	17 (19.3%)	2 (15.4%)	15 (20.0%)	1.000
Risk factors for VTE and comorbidities				
Previous PE	14 (17.3%) (*n = *81)	4 (44.4%) (*n = *9)	10 (13.9%) (*n = *72)	0.044
Cancer	18 (21.7%) (*n = *83)	4 (44.4%) (*n = *9)	14 (18.9%) (*n = *74)	0.097
Chronic cardiac or pulmonary disease	21 (23.9%)	1 (7.7%)	20 (26.7%)	0.177
CKD with GFR 15 mL/min/1,73 m^2^	3 (3.7%) (*n = *82)	1 (11.1%) (*n = *9)	2 (2.7%) (*n = *73)	1.000
Arterial hypertension	58 (68.2%) (*n = *85)	5 (50.0%) (*n = *10)	53 (70.7%) (*n = *75)	0.277
Coronary artery disease	12 (14.6%) (*n = *82)	0 (0.0%) (*n = *9)	12 (16.4%) (*n = *72)	0.343
Chronic cardiac or pulmonary disease	21 (23.9%)	1 (7.7%)	20 (26.7%)	0.177
Bleeding history	2 (2.5%) (*n = *81)	0 (0.0%) (*n = *9)	2 (2.8%) (*n = *72)	1.000
Symptoms and clinical findings on admission				
Dyspnea NYHA IV	12 (14.0%) (*n = *86)	1 (7.7%) (*n = *13)	11 (15.1%) (*n = *73)	0.462
Heart rate ≥ 110 bpm	23 (28.8%) (*n = *80)	4 (33.3%) (*n = *12)	19 (27.9%) (*n = *68)	0.736
Blood pressure < 100 mmHg	12 (15.8%) (*n = *77)	5 (41.7%) (*n = *12)	7 (10.8%) (*n = *65)	0.017
Blood pressure < 90 mmHg	6 (7.9%) (*n = *76)	5 (41.7%) (*n = *12)	1 (1.6%) (*n = *64)	< 0.001
CPR on admission	8 (9.8%) (*n = *82)	7 (53.8%) (*n = *13)	1 (1.4%) (*n = *69)	< 0.001
SpO_2_ < 90%	26 (55.3%) (*n = *47)	3 (60.0%) (*n = *5)	23 (54.8%) (*n = *42)	1.000
Shock parameters	10 (11.4%)	7 (53.8%)	3 (4.0%)	< 0.001
NIV on admission	6 (7.6%) (*n = *79)	3 (27.3%) (*n = *11)	3 (4.4%) (*n = *68)	< 0.001
Invasive mechanical ventilation	7 (8.9%) (*n = *79)	33 (27.3%) (*n = *11)	4 (5.9%) (*n = *68)	< 0.001
Imaging on admission				
CT on admission	84 (95.5%)	13 (100.0%)	71 (94.7%)	1.000
CT: RV/LV > 1	62 (84.9%) (*n = *73)	7 (70.0%) (*n = *10)	55 (87.3%) (*n = *63)	0.168
Echo: RVD	34 (66.7%) (*n = *51)	2 (100.0%) (*n = *2)	32 (65.3%) (*n = *49)	0.547
Laboratory values on admission				
Median lactate in mmol/L and IQR	3.2 (2.1–7.9) (*n = *49)	6 (4.6–10.3) (*n = *9)	1.5 (1–2.4) (*n = *40)	0.003
Median hemoglobin in g/dl and IQR	12.5 (10.9–14.2) (*n = *85)	10.9 (10.2–13.2) (*n = *11)	13.25 (11.9–14.7) (*n = *74)	0.034
Elevated cardiac biomarker	66 (91.7%) (*n = *72)	8 (100.0%) (*n = *8)	58 (90.6%) (*n = *64)	1.000
Risk stratification				
sPESI points ≥ 1	63 (71.6%)	11 (84.6%)	52 (69.3%)	0.334
ESC algorithm 2019: low risk	2 (2.3%)	0 (0.0%)	2 (2.7%)	-
ESC algorithm 2019: intermediate–low risk	39 (44.3%)	2 (15.4%)	37 (49.3%)	-
ESC algorithm 2019: intermediate–high risk	37 (42.0%)	4 (30.8%)	33 (44.0%)	-
ESC algorithm 2019: high risk	10 (11.4%)	7 (53.8%)	3 (4.0%)	-
Treatment in-hospital				
Systemic full-dose thrombolysis	7 (8.0%) (*n = *87)	4 (30.8%)	3 (4.1%) (*n = *74)	0.145
Systemic half-dose thrombolysis	1 (1.1%) (*n = *87)	0 (0.0%)	1 (1.4%) (*n = *74)	1.000
Percutaneous thrombectomy	1 (1.1%) (*n = *87)	0 (0.0%)	1 (1.4%) (*n = *74)	1.000
Surgical embolectomy	2 (2.3%) (*n = *87)	1 (7.7%)	1 (1.4%) (*n = *74)	0.923
ICU admission	63 (73.3%) (*n = *86)	10 (83.3%) (*n = *12)	53 (71.6%) (*n = *74)	0.502
Complications during in-hospital course				
Pneumonia	29 (34.1%) (*n = *85)	4 (30.8%)	25 (34.7%) (*n = *72)	1.000
Use of catecholamines	23 (26.1%)	11 (84.6%)	12 (16.0%)	< 0.001
Non-Invasive Ventilation	10 (11.4%)	2 (15.4%)	8 (10.7%)	0.638
Intubation	18 (20.7%) (*n = *87)	8 (61.5%)	10 (13.5%) (*n = *74)	0.001
Stroke	4 (4.6%) (*n = *87)	0 (0.0%)	4 (5.4%) (*n = *74)	1.000
Paradoxical embolism	3 (3.4%) (*n = *87)	1 (7.7%)	2 (2.7%) (*n = *74)	0.388
CPR in clinical course	6 (31.6%) (*n = *19)	6 (100.0%) (*n = *6)	0 (0.0%) (*n = *13)	< 0.001
Bleeding: severe bleeding	1 (1.1%) (*n = *87)	1 (7.7%)	0 (0.0%) (*n = *74)	1.000
Bleeding: moderate bleeding	6 (6.9%) (*n = *87)	3 (23.1%)	3 (4.1%) (*n = *74)	0.001
Cause of death during in-hospital stay due to PE event				
Acute PE event	12 (14.1%) (*n = *85)	12 (92.3%)	0 (0.0%) (*n = *72)	< 0.001
Bleeding	1 (1.2%) (*n = *85)	1 (7.7%)	0 (0.0%) (*n = *72)	1.000

Due to observational character of the study and potential transfer from other hospitals, information might not be available for all patients

### PERT era: first contact and its composition in a multidisciplinary team

**Fig. 1:**
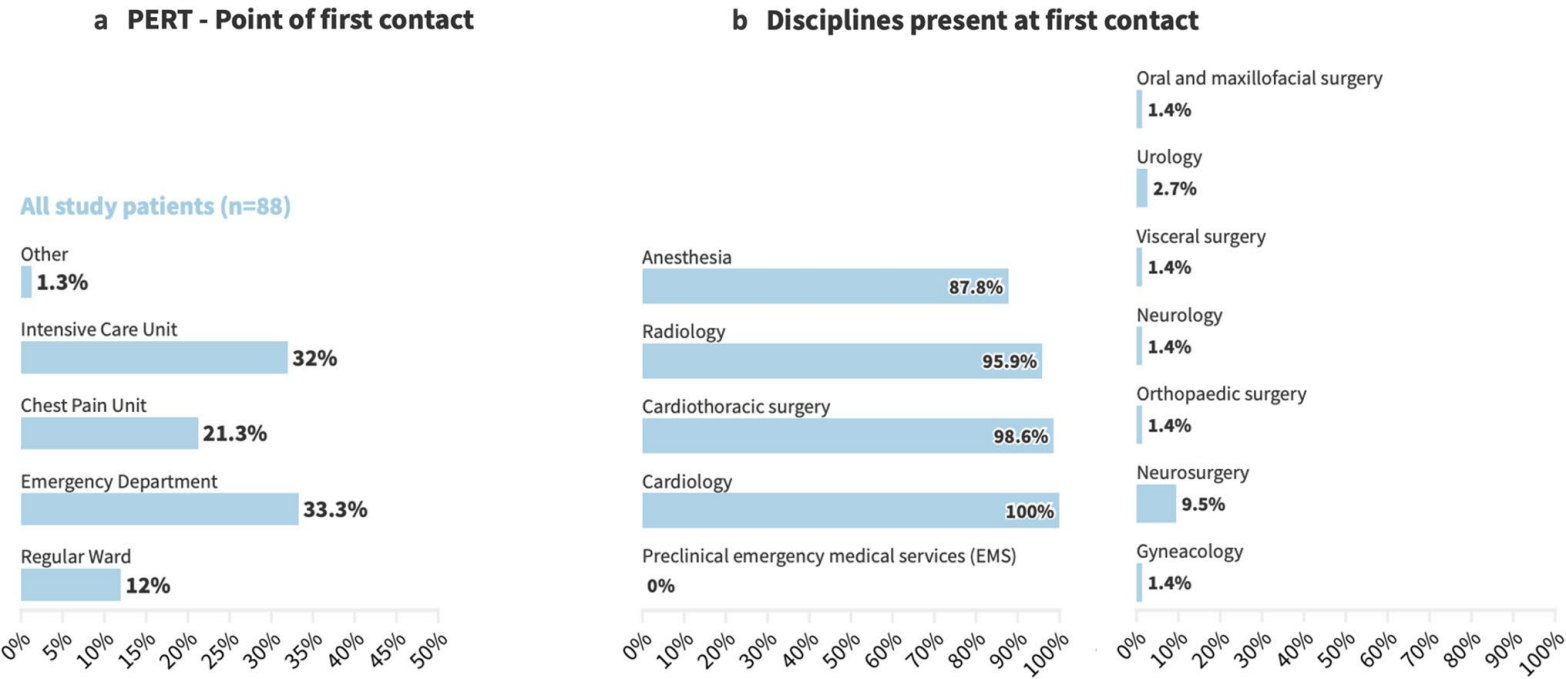
**a** Point of first PERT contact and **b** disciplines presented in first PERT

First PERT contact originated mainly by emergency departments (33.3%) and intensive care unit (30.0%), followed by chest pain unit (21.3%) and regular ward (12.0%) (Fig. 1a). The participation rate of specialties demonstrated that cardiologists (100%) or cardiac/vascular surgeons (98.6%) were included in almost all PERT activations, followed by radiologists (95.9%), and anesthesiologists (87.8%). Further disciplines were present in a few cases, as summarized in Fig. 1b, usually in the role of the physician in charge of the primary disease.

### PERT era: treatment and outcomes

Most patients (87.5%) received intravenous heparin treatment only. Systemic thrombolysis was administered in 8 (9.1%) patients; one patient received half-dose systemic thrombolysis. Overall, one patient was treated with percutaneous local thrombectomy, and surgical embolectomy was performed in two patients. From the time of PERT contact, patients received reperfusion treatment in a median of 25 min (IQR 9–69).

In a logistic regression analysis, adjusted for age and sex, the most important predictors for in-hospital mortality are the following: Prior pulmonary embolism in medical history, malignancy, chronic kidney disease, and according to the clinical presentation: mild hypotension, shock, need for catecholamines, need for mechanical ventilation and cardiac arrest/cardiopulmonary resuscitation (Table 2).

**Table 2:** Associations of parameters in the PERT era with in-hospital mortality—multivariate regression (adjusted for age and sex)

	OR (95% CI)	*p* value
Prior PE	5.174 (1.099–24.360)	0.038
Malignancy	5.067 (1.049–24.475)	0.043
Atrial fibrillation	10.022 (1.515–66.299)	0.017
Chronic kidney disease	1.635 (1.004–2.663)	0.048
Blood pressure < 100 mmHg	6.772 (1.591–28.827)	0.010
Blood pressure < 90 mmHg	46.662 (4.488–485.125)	0.001
Lactate > 2 mmol/l	6.581 (2.691–16.091)	< 0.001
CPR on admission	190.644 (11.381–3193.468)	< 0.001
ESC algorithm 2019	8.483 (2.584–27.847)	< 0.001
Use of catecholamines	38.130 (6.406–226.947)	< 0.001
Mechanical ventilation	10.206 (2.760–37.737)	< 0.001
Bleeding	3.085 (1.291–7.368)	0.011

### PERT era vs. pre-PERT era: comparison of two time periods

**Fig. 2:**
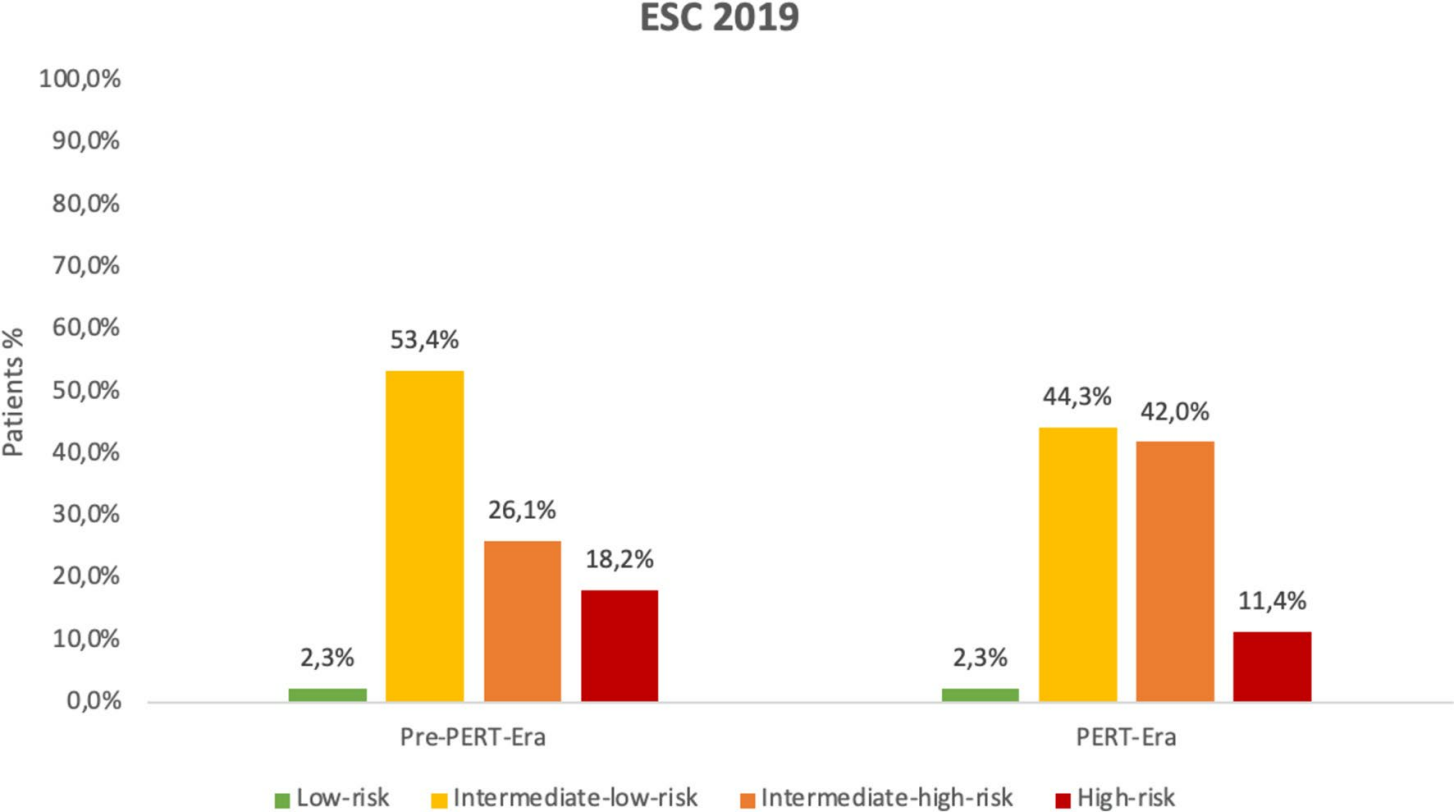
Risk stratification of PE according to ESC classification

In total, 124 patients treated with acute PE in an intermediate-care to intensive care unit were chosen between 2017 and 2018. After propensity matching by including age, sex, sPESI points, and ESC 2019 algorithm, 88 patients with acute PE were identified for the final analysis to compare pre-PERT era with PERT era (Fig. S2). When comparing patients’ characteristics of the pre-PERT era with the PERT era, more patients were classified as sPESI ≥ 1 and as high-risk according to ESC 2019 guidelines in the pre-PERT era (78.4% and 18.2%) opposed to the PERT era (71.6 and 11.4%) (Fig. 2). Patients in the pre-PERT era had more often a bleeding history and a lower hemoglobin level on admission. Moderate and severe bleeding were more frequent in the pre-PERT era, most likely linked to the more frequent use of rescue reperfusion options, such as systemic thrombolysis, surgical, or percutaneous thrombectomy (Table 3). Advanced reperfusion therapies such as surgical embolectomy (*n = *8) were performed in the pre-PERT era also in low-risk (*n = *1, 12.5%) and intermediate–low-risk patients with acute PE (*n = *4, 50%), whereas in the PERT era, surgical embolectomy was performed in two cases only (one case in an intermediate–low-risk patient and one case in a high-risk PE patient). Regarding the time from diagnosis of PE to reperfusion, patients received reperfusion treatment slightly faster in the PERT era as opposed to the pre-PERT era (100 [55–246] minutes vs. 120 [120–219]; *p = *0.121).

**Table 3:** Main differences in baseline characteristics and outcomes of patients in the PERT era versus the pre-PERT era

	All study patients (*n = *176)	PERT era (*n = *88)	Pre-PERT era (*n = *88)	*p* value
Sex (male)	89 (50.6%)	45 (51.1%)	44 (50.0%)	1.000
Median age and IQR	67 (58–77)	68 (58–79)	67 (56–77)	0.652
Age above 80	26 (14.8%)	17 (19.3%)	9 (10.2%)	0.136
Median hospitalization days and IQR	11 (7–20) (*n = *170)	9 (5–16) (*n = *82)	13.5 (9–26)	0.011
Risk factors for VTE and comorbidities				
Previous PE	19 (11.4%) (*n = *167)	14 (17.3%) (*n = *81)	5 (5.8%) (*n = *86)	0.027
Recent surgery	65 (38.7%) (*n = *168)	18 (22.0%) (*n = *82)	47 (54.7%) (*n = *86)	< 0.001
Arterial hypertension	103 (59.9%) *n = *172)	58 (68.2%) (*n = *85)	45 (51.7%) (*n = *87)	0.030
Bleeding history	13 (7.8%) (*n = *167)	2 (2.5%) (*n = *81)	11 (12.8%) (*n = *86)	0.018
Symptoms and clinical findings on admission				
Heart rate ≥ 110 bpm	49 (32.5%) (*n = *151)	23 (28.8%) (*n = *80)	26 (36.6%) (*n = *71)	0.736
Blood pressure < 100 mmHg	23 (15.5%) (*n = *148)	12 (15.6%) (*n = *77)	11 (15.5%) (*n = *71)	1.000
Blood pressure < 90 mmHg	12 (8.2%) (*n = *147)	6 (7.9%) (*n = *76)	6 (8.5%) (*n = *71)	1.000
CPR on admission	20 (12.2%) (*n = *164)	8 (9.8%) (*n = *82)	12 (14.6%) (*n = *82)	0.475
SpO_2_ < 90%	36 (56.3%) (*n = *64)	26 (55.3%) (*n = *47)	10 (58.8%) (*n = *17)	1.000
Shock parameters	25 (14.2%)	10 (11.4%)	15 (17.0%)	0.388
Imaging on admission				
CT on admission	163 (93.1%) (*n = *175)	84 (95.5%)	79 (90.8%) (*n = *87)	0.248
CT RV/LV > 1	106 (75.2%) (*n = *141)	62 (84.9%) (*n = *73)	44 (64.7%) (*n = *68)	0.006
RVD Echo	11 (73.3%) (*n = *15)	10 (76.9%) (*n = *13)	1 (50.0%) (*n = *2)	0.476
Laboratory values on admission				
Median lactate in mmol/L and IQR	2.0 (1.1–3.4) (*n = *111)	1.7 (1.0–3.9) (*n = *49)	2.3 (1.1–3.3) (*n = *62)	0.603
Median hemoglobin in g/dl and IQR	12.2 (10.9–14.2) (*n = *85)	13.1 (11.6–14.7) (*n = *85)	11.2 (9.5–13) (*n = *83)	< 0.001
Elevated cardiac biomarker	125 (89.3%) (*n = *140)	66 (91.7%) (*n = *72)	59 (86.8%) (*n = *68)	0.418
Treatment in-hospital				
Systemic full-dose thrombolysis	19 (11.0%) (*n = *172)	7 (8.0%) (*n = *87)	12 (14.1%) (*n = *85)	0.181
Systemic half-dose thrombolysis	1 (0.6%) (*n = *172)	1 (1.1%) (*n = *87)	0 (0.0%) (*n = *85)	1.000
Percutaneous thrombectomy	7 (4.1%) (*n = *172)	1 (1.1%) (*n = *87)	6 (7.1%) (*n = *85)	0.044
Surgical embolectomy	8 (4.7%) (*n = *172)	2 (2.3%) (*n = *87)	6 (7.1%) (*n = *85)	0.191
Complications during in-hospital course				
Pneumonia	72 (41.9%) (*n = *172)	29 (34.1%) (*n = *85)	43 (49.4%) (*n = *87)	0.046
Use of catecholamines	62 (35.8%) (*n = *173)	23 (26.1%)	39 (45.9%) (*n = *85)	0.007
Non-Invasive Ventilation	25 (14.4%) (*n = *174)	10 (11.4%)	15 (17.4%) (*n = *86)	0.285
Intubation	46 (26.4%) (*n = *174)	18 (20.7%) (*n = *87)	28 (32.2%) (*n = *87)	0.121
CPR in clinical course	16 (15.1%) (*n = *106)	6 (31.6%) (*n = *19)	10 (11.5%) (*n = *87)	0.038
Bleeding: severe bleeding	13 (7.5%)	1 (1.1%)	12 (13.8%)	0.021
Bleeding: moderate bleeding	16 (9.2%) (*n = *174)	6 (6.9%) (*n = *87)	10 (11.5%) (*n = *87)	0.311
Cause of death during in-hospital stay due to PE event				
Acute PE event	32 (16.7%) (*n = *171)	12 (14.1%) (*n = *85)	20 (23.3%) (*n = *86)	0.322
Bleeding	2 (1.2%) (*n = *171)	1 (1.2%) (*n = *85)	1 (1.2%) (*n = *86)	1.000
Sepsis	2 (1.2%) (*n = *171)	0 (0.0%) (*n = *85)	2 (2.3%) (*n = *86)	0.548

Due to observational character of the study and potential transfer from other hospitals, information might not be available for all patients

Further differences are summarized in Table 3. A considerably higher all-cause mortality (31.8% vs. 14.8%) and PE-related mortality rate (22.7% vs. 13.6%) was observed in patients in the pre-PERT era compared to the PERT era. To further adjust for PE severity, multivariable regression analyses included parameters such as the admission status on the intensive care unit and shock parameters. This analysis revealed that PE management in the PERT era was associated with a lower risk of all-cause mortality (OR, 0.35 [95%CI 0.15–0.84]; *p = *0.018). Regarding PE-related mortality, a tendency, but no significant odds were found, to reduced PE-related mortality events (OR, 0.57 [95%CI 0.22–1.146; *p = *0.241) in the PERT era compared to patients before the PERT era (Table 3).

## Discussion

Pulmonary embolism (PE) is a significant cause of morbidity and mortality worldwide. While national and international guidelines for diagnosing and managing PE have been well-established, implementing a specialized pulmonary embolism response team (PERT) remains a matter of debate [3]. Another example of a specialized disease response team is the heart team with increasing acceptance worldwide for the multidisciplinary management of patients with cardiovascular disease [11]. PERT aims to provide rapid diagnosis and treatment of PE, including advanced imaging, risk stratification, and selection of appropriate therapy. PERT has been shown to reduce mortality rates and hospital length of stay, making it an essential addition to any healthcare system [12, 13]. However, the impact of PERT on the outcome of PE remains unclear due to the fact that prospective studies with clear outcomes are still missing [6, 14].

Even if the ESC underlines the importance of set-up, a multidisciplinary team and a program for managing high- and intermediate–high-risk PE with a class IIa recommendation, reports from Europe are only limited to two single-centre experiences from Poland and France [15, 16]. The current study is the first report demonstrating data and knowledge from implementing a PERT in a German center. In line with a recent meta-analysis, the composition of PERT in Germany is equally distributed with cardiologists and cardiac/vascular surgeons in almost all PERT cases, followed by a radiologist and anesthesiologists or intensivist [6]. The commonly applied risk stratifications used in the PERT era showed the expected results. Non-survivors had higher lactate levels, lower hemoglobin, and higher sPESI score and showed more severe ESC algorithm 2019 classes.

When comparing the PERT era with the pre-PERT era at our institution, the most important finding is an overall reduction of in-hospital mortality in the PERT era.

Several explanations for this reduction in mortality can be discussed. The higher incidence of bleeding history in the pre-PERT collective combined with the lower median hemoglobin levels might have influenced the therapeutic decisions made by the attending physicians. Although the major bleeding as a cause of death as well as shock parameters and blood pressure did not differ considerably between both groups, a significant higher rate of catecholamine use in the pre-PERT era was observed. In this context, a trend toward percutaneous thrombectomy and surgical embolectomy could be a possible explanation to avoid thrombolysis associated bleeding complications [17]. In contrast, the rate of CT:RV/LV ratio > 1 was significantly higher in PERT era. The lower rate of embolectomy could be explained by the hypothesis, that in the pre-PERT era, decisions were often made by a single discipline. Thus, the decisions may favor well-known treatment strategies of the single discipline, in which the patient was primary admitted, whereas in a multidisciplinary team, the indication and the type of reperfusion are discussed more intensively.

In the Multicenter Emergency Medicine Pulmonary Embolism in the Real World Registry (EMPEROR), 2% of PE overall and 9% of PE with high-risk PE were treated with systemic thrombolysis [18], which is in line with results in our center with 8% of all patients with PE and a PERT protocol. Since PERT is implemented in several countries and hospitals, the overuse of invasive techniques was a main matter of concern [4]. Several single-center studies from the United States found no significant reduction in mortality, but at the same time a trend toward more intensified therapies [19–21]. This was not the case in our cohort, more patients in the pre-PERT era received advanced reperfusion therapies as systemic full-dose thrombolysis or surgical embolectomy than patients in the PERT era, which could potentially explain the higher rate of bleeding complications in the pre-PERT era. Additionally, we found that these advanced reperfusion therapies were performed in low-risk and intermediate–low-risk patients with PE significantly more frequently in the pre-PERT era than in the PERT era. Presumably, in a multidisciplinary approach, a team includes endovascular interventionalists, surgeons, and non-invasive physicians, who weigh up advantages and disadvantages and guiding the optimal treatment according to risk stratification evidence. Previously, a single-center study from the United States showed that a dedicated PERT results in the efficient delivery of care and excellent outcomes, which is in line with our findings showing a rapid time to initiate treatment in 25 min after the multidisciplinary discussion [22]. According to the local standard operating procedure, all patients were treated with unfractionated heparin (UFH) during the initial phase. The UFH dosage was given to patient individual and was not recorded in this study. Additionally, the long-term anticoagulation regimes were not part of our study protocol and thus not documented.

A further aspect that must be considered is that our data demonstrated a higher rate of pneumonia and sepsis in patients with acute PE in the pre-PERT era than patients treated in the PERT era. This finding emphasizes that the team approach promotes consensus and provides a unified, reasoned plan for the individual patient, improving efficiency over the traditional practice of independently consulting numerous subspecialty physicians. In this context, although the prognostic value of right ventricular dilation in computed tomography is established to guide risk-adjusted management strategy for acute PE, it is known that additional information about the tricuspid annular plane systolic excursion in echocardiography can identify patients at higher risk for an adverse outcome compared to single parameters of RV enlargement [23]. Before the implementation of PERT in Mainz, only a minority of patients received echocardiography, indicating on the one hand that further aspects of RV dysfunction were not included in the decision-making process, and on the other hand, suggesting that the consultation of a cardiologist has probably not taken place in every case.

The present study has limitations that need consideration: first, our data are limited to a single center including the resources of its electronic medical records. Second, some pre- to post-PERT implementation changes may be partially explained by different cohorts picked from ICD codes, which were tried to balance by propensity score matching. If systemic thrombolysis failed or is contraindicated, alternative reperfusion strategy was recommended with surgical thrombectomy in line with the 2014 ESC guidelines on pulmonary embolism. In the meantime, the 2019 ESC guidelines on pulmonary embolism expand the recommendations toward catheter-directed treatment (CDT) like catheter-based thrombectomy or catheter-directed low-dose thrombolysis as an alternative [7, 8]. Following the paradigm shift, further studies might elucidate the role of CDT in different indications. The role of PERT becomes even more important in relation to technical improvements and updated guidelines regarding CDT strategies. These new technologies offer additional therapeutic options while bringing along their own risk–benefit relations. This should be discussed in a PERT to identify patients with the highest expected benefits and to prevent an intention-to-intervene bias.

In conclusion, implementing PERT was associated with less-invasive therapeutic strategies such as systemic thrombolysis or surgical embolectomy, presumably followed by reduced bleeding complications, and decreased all-cause and PE-related mortality. With its ability to cross disciplines and quickly mobilize resources for decompensating patients, a multidisciplinary team can be seen as crucial for managing patients with complex and higher-risk PE.

### Supplementary Information

Below is the link to the electronic supplementary material.

Supplementary file1 (DOCX 98 kb)

## Data Availability

The data underlying this article are available in the article and in its online supplementary material.
