# Supplementary figures and images for: A multidisciplinary pulmonary embolism response team (PERT): first experience from a single center in Germany
Source: Clin Res Cardiol. 2023 Dec 19;113(4):581–90. doi: 10.1007/s00392-023-02364-4 (PMC10954947; doi:10.1007/s00392-023-02364-4)

**Supplementary Figures**

**
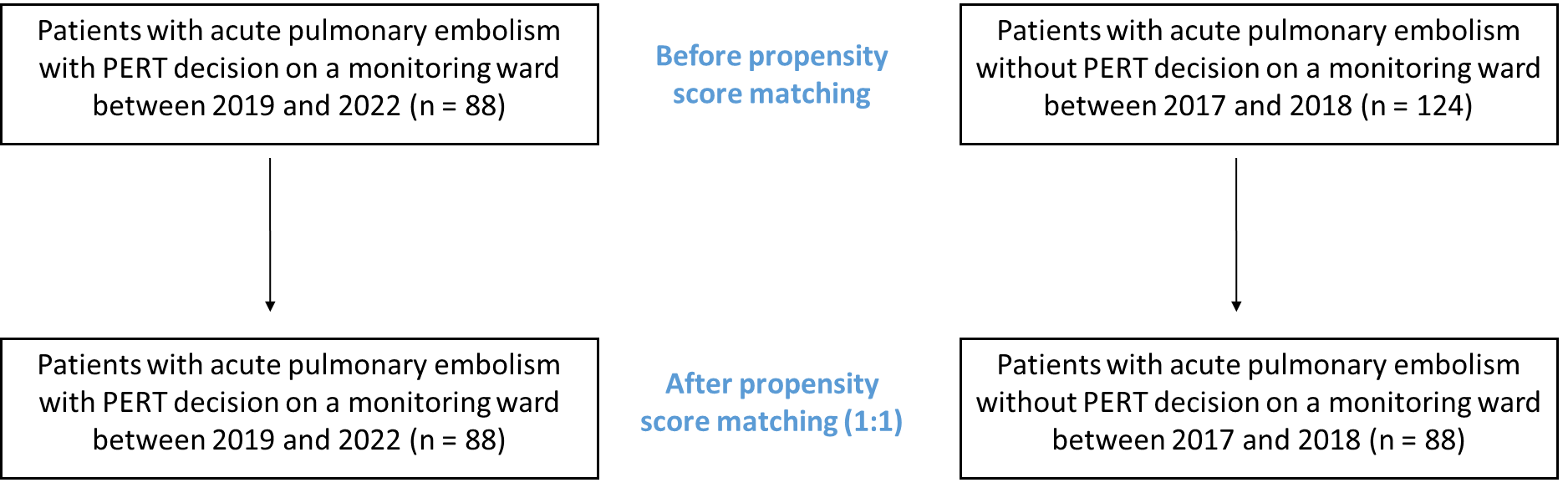
**

**Fig. S1** Study flow chart

**
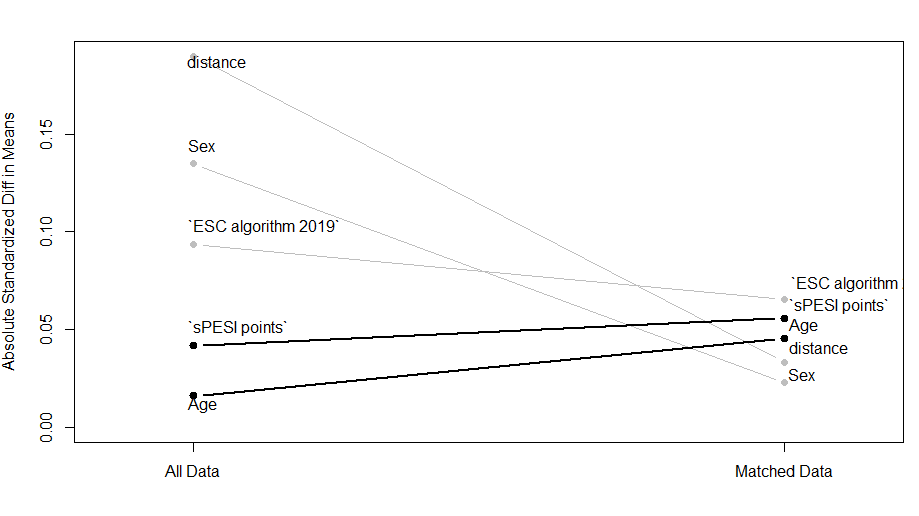
**

**Fig. S2** Balance in propensity score matching

Supplement: Supplementary file 1 — Supplementary file1 (DOCX 98 kb) [file 392_2023_2364_MOESM1_ESM.docx]
